# Concurrent mapping of multiple epigenetic marks and co-occupancy using ACT2-seq

**DOI:** 10.1186/s13578-021-00711-4

**Published:** 2021-12-04

**Authors:** Benjamin Carter, Wai Lim Ku, Joe Pelt, Keji Zhao

**Affiliations:** Laboratory of Epigenome Biology, Systems Biology Center, National Heart, Lung and Blood Institute, NIH, Bethesda, MD 20892 USA

**Keywords:** Co-occupancy, Co-enrichment, ACT-seq, Tagmentation, Epigenetic marks

## Abstract

**Background:**

Genome-wide profiling of epigenetic marks is a core technology in molecular genetics. Co-occupancy of different epigenetic marks or protein factors at the same genomic locations must often be inferred from multiple independently collected data sets. However, this strategy does not provide direct evidence of co-enrichment in the same cells due to the existence of cellular heterogeneity. To address this issue, we have developed a technique termed ACT2-seq that is capable of concurrently profiling multiple epigenetic marks in a single biological sample. In addition to reducing the numbers of samples required for experiments, ACT2-seq is capable of mapping co-occupancy of epigenetic factors on chromatin. This strategy provides direct evidence of co-enrichment without requiring complex single-molecule, single-cell, or magnetic bead-based approaches.

**Results:**

We concurrently profiled pairs of two epigenetic marks using ACT2-seq as well as three marks in individual samples. Data obtained using ACT2-seq were found to be reproducible and robust. ACT2-seq was capable of cleanly partitioning concurrently mapped data sets that exhibited distinct enrichment patterns. Using ACT2-seq, we identified distinct relationships between co-occupancy of specific histone modifications and gene expression patterns.

**Conclusions:**

We conclude that ACT2-seq presents an attractive option for epigenomic profiling due to its ease of use, potential for reducing sample and sequencing costs, and ability to simultaneously profile co-occupancy of multiple histone marks and/or chromatin-associated proteins.

**Supplementary Information:**

The online version contains supplementary material available at 10.1186/s13578-021-00711-4.

## Background

Cellular processes such as differentiation and responses to environmental stimuli often involve persistent changes in gene expression. In eukaryotic cells, these processes are facilitated by chromatin-based features including covalent histone modifications, chromatin accessibility, and DNA methylation. Such features are referred to as “epigenetic marks” and are deposited and recognized by chromatin-associated “writers” and “readers” [1–3]. In addition to binding chromatin-associated machinery, epigenetic marks can influence gene expression through other mechanisms including modifying the mechanical properties of chromatin [4] or promoting changes in higher-order chromatin organization [5–7].

The most widely adopted method for mapping epigenetic marks has been chromatin immunoprecipitation followed by sequencing (ChIP-seq), in which antibodies that target the epigenetic mark of interest are used to precipitate and isolate chromatin fragments that bear the mark [8, 9]. DNA is then purified from the enriched chromatin fragments and sequenced using high-throughput sequencing technologies. The resulting sequence reads are mapped to the organism’s reference genome to generate a genome-wide enrichment profile for the targeted epigenetic mark. Data obtained using ChIP-seq is often robust and reproducible, making it an invaluable tool in the field of molecular genetics.

Due to the important roles of epigenetic marks in controlling transcription and chromatin organization, it is valuable to understand which epigenetic factors co-occupy a gene promoter or other regulatory region. Identifying co-occupancy of chromatin features, such as the binding of transcription factors along with the presence of specific histone modifications, provides important mechanistic insights into chromatin-based phenomena. For example, co-occupancy of two chromatin features at a gene regulatory region raises the possibility that they function cooperatively or synergistically and can clarify models of the mechanisms of gene regulation. Co-occupancy of epigenetic marks and/or chromatin-binding proteins is often inferred using their enrichment at the same genomic locations from independent epigenomic datasets [10, 11]. However, most available methods for profiling epigenomic marks provide a single aggregate signal compiled from thousands or millions of cells. Enrichment profiles generated from such samples are not sufficient to establish that co-occupancy occurs due to the extensive effects of cell-to-cell heterogeneity in epigenetic states, which is present even among highly similar and clonally derived cells [12]. Thus, genomic regions exhibiting enrichment for multiple epigenetic marks in different samples can arise from heterogeneous sub-populations of cells in which the two marks are seldom or never present at the same time in the same cells [12].

We previously developed a chromatin profiling method, antibody-guided chromatin tagmentation sequencing (ACT-seq), based on the DNA transposition activity of Tn5 transposase [13]. In ACT-seq, Tn5 is fused to Protein A to facilitate antibody-based binding of the transposase to targeted epigenetic marks and transcription factors. This allows chromatin fragmentation and adapter ligation to be performed by the Tn5 enzyme during a single experimental step specifically at genomic locations enriched for the epigenetic mark of interest. ACT-seq dramatically reduces the need for experimental optimization relative to ChIP-seq and its derivative methods. In this study, we present an expanded and streamlined version of ACT-seq known as antibody-guided chromatin tagmentation for two factors followed by sequencing (ACT2-seq, ACT2). Like ACT-seq, ACT2-seq can be completed in one day of bench work and does not require expensive reagents such as magnetic beads, sonicators, or library preparation kits. ACT2-seq makes use of barcoded transposase adapters to enable concurrent profiling of two epigenetic marks in a single biological sample, which can reduce the numbers of samples required for experiments. Importantly, this barcoding strategy simultaneously probes for co-enrichment of epigenetic marks, which previously required technically challenging or laborious approaches involving single cells, single-molecule fluorescence, or sequential ChIP [14, 15]. This approach provides direct evidence of co-occupancy of epigenetic marks, avoiding the issue of cellular heterogeneity that confounds interpretation of co-enrichment using bulk-cell ChIP-seq data. We conclude that these features make ACT2 an appealing option for whole-genome profiling of epigenetic factors.

## Results

**Fig. 1:**
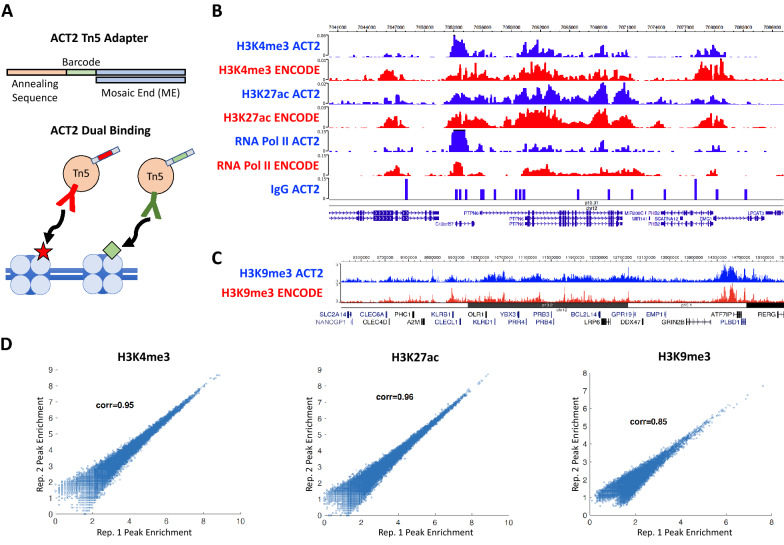
Enrichment profiles generated using ACT2-seq are robust and reproducible. **A** Depiction of the ACT2 adapter design showing its three constitutive regions (top). Illustration of the concept of dual binding in ACT2 (bottom). Each antibody targets a distinct epigenetic factor and facilitates incorporation of a barcoded adapter via the associated Tn5 transposase subunit. **B** Genome browser snapshot of a representative genomic region enriched for “active” chromatin marks. ENCODE-validated ChIP-seq data sets are provided for comparison. IgG serves as the non-specific control for enrichment. **C** Genome browser snapshot of a representative genomic region enriched for H3K9me3. ENCODE-validated ChIP-seq data are provided for comparison. **D** Scatter plots examining peak enrichment correlation between two biological replicates of the same samples. Each data point represents the read density of a single enriched region

ACT2 uses a fusion of Protein A with Tn5 transposase (PA-Tn5), which was previously introduced for use with ACT-seq [13]. With ACT2, however, the oligonucleotide transposase adapters are barcoded to enable different antibodies to be used in the same biological sample (Fig. 1A, top). Separate aliquots of PA-Tn5 enzyme are bound to discrete combinations of adapters and antibodies, which allows for concurrent binding of multiple labeled PA-Tn5 complexes to chromatin (Fig. 1A, bottom). After the binding steps, any unbound PA-Tn5 complexes are washed away, and tagmentation is initiated to capture signals specifically arising from bound genomic locations. This strategy enables the DNA fragments generated by ACT2 to be sorted into separate signals based on the adapter barcodes present on the fragment.

To examine whether data obtained using the modified ACT2 adapters were robust and reproducible, we probed multiple epigenetic factors in cultured human GM12878 lymphoblast cells: trimethylation of lysine 4 on histone H3 (H3K4me3), acetylation of lysine 27 on histone H3 (H3K27ac), trimethylation of lysing 9 on histone H3 (H3K9me3), and RNA Polymerase II (RNA Pol II). These factors were selected to profile both transcriptionally repressed and active genomic regions as well as a chromatin-associated protein complex. Immunoglobulin G (IgG) was included as a control for non-specific binding and enzyme activity. Visual inspection of the peak patterns produced by ACT2 at genomic regions enriched for the “active” chromatin marks revealed a high level of similarity to ENCODE-validated ChIP-seq data sets (Fig. 1B). In comparison, the IgG-bound complex produced sparse and intermittent signals with little to no peak formation, indicating that the enrichment patterns detected using ACT2 arise from specific antibody binding to target sites. We similarly examined genomic regions enriched for H3K9me3 and observed strong similarities between the enrichment patterns obtained using ACT2 and those from the ENCODE ChIP-seq data (Fig. 1C). To further examine the robustness of data obtained using ACT2, we computationally identified regions of enrichment (“peaks”) for each biological replicate of H3K4me3, H3K27ac, and H3K9me3. The read densities within these peaks were found to be highly similar between biological replicates of the same treatment: over 85% correlation in each case (Fig. 1D).

**Fig. 2:**
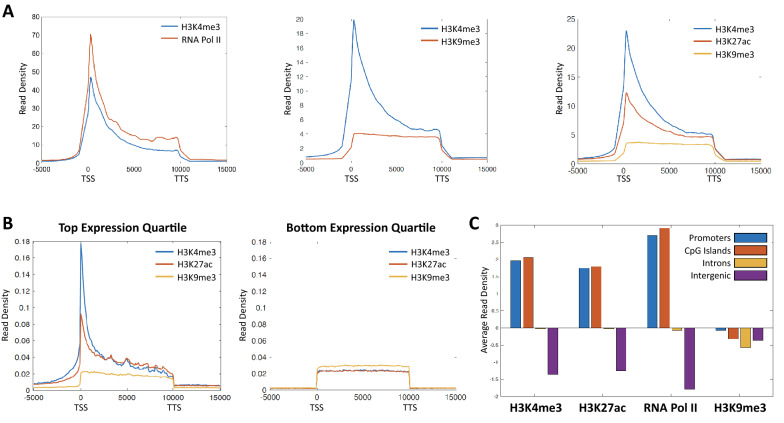
ACT2-seq efficiently partitions co-profiled data sets across various genomic elements. **A** Metagene profiles of average read density within annotated genes for samples in which the indicated epigenetic factors were mapped concurrently. TSS: transcription start site. TTS: transcription termination site. Gene lengths have been scaled to 10 kb to facilitate alignment. **B** Metagene profiles as in panel A but for the sets of genes representing the top and bottom expression quartiles. **C** Comparison of mean read densities within a variety of genomic region types for the indicated epigenetic marks. Read densities were normalized using the length of each region

The ACT2 barcoding strategy (Fig. 1A) enables concurrent profiling of multiple epigenetic marks in a single biological sample. To investigate whether ACT2 is capable of efficiently partitioning the multiple data sets generated from a single sample, we examined enrichment patterns for epigenetic factors that were mapped concurrently. Many previous studies have identified distinct enrichment signatures for specific “active” epigenetic marks at transcription start sites (TSSs) and enhancers linked to highly expressed genes [16–18]. Consistent with these studies, we observed strong enrichment of both H3K4me3 and RNA Pol II at gene TSSs when both marks were mapped concurrently in the same ACT2 sample (Fig. 2A, left). In contrast, co-profiling of H3K4me3 alongside the repressive histone modification H3K9me3 resulted in strikingly different patterns of average enrichment (Fig. 2A, middle). Further, we found that the distinct H3K9me3 enrichment profile was maintained even when this mark was co-profiled alongside two active histone modifications in the same sample (Fig. 2A, right). These results indicate that ACT2 is capable of partitioning data sets that exhibit disparate enrichment patterns from the same biological sample.

To further examine whether ACT2 efficiently resolves co-profiled data sets, we visualized enrichment of these factors across different classes of genes and genomic elements. Using publicly available RNA-seq data from the same cell type [19], we identified gene sets corresponding to the top and bottom quartiles of expression. We examined average enrichment of three co-profiled epigenetic marks (Fig. 2A, right) at these categories of genes and observed pronounced enrichment of the active histone marks at active TSSs without concurrent H3K9me3 enrichment (Fig. 2B, left). In contrast, genes in the bottom quartile of expression were not enriched for the active marks at the TSSs, suggesting an expression-dependent role for these factors. Based on these results, we predicted that H3K9me3 would exhibit distinct enrichment patterns relative to the active marks across other classes of genomic elements as well. To test this prediction, we calculated relative enrichment across a variety of genomic elements including promoters, CpG islands, introns, and intergenic regions. For the active epigenetic marks H3K4me3, H3K27ac, and RNA Pol II, we observed elevated enrichment at gene promoters and at CpG islands, which are often associated with proximal gene promoters in humans [17, 20] (Fig. 2C). Consistent with our conjecture, we did not observe enrichment of H3K9me3 at these elements, which supports the distinct distribution of H3K9me3 relative to the active marks. For comparison, all of the epigenetic marks we examined were relatively sparsely enriched at introns and intergenic regions. Taken together, these analyses support the robustness of ACT2 co-profiling for epigenetic marks with distinct enrichment patterns at various classes of genes and genomic elements.

**Fig. 3:**
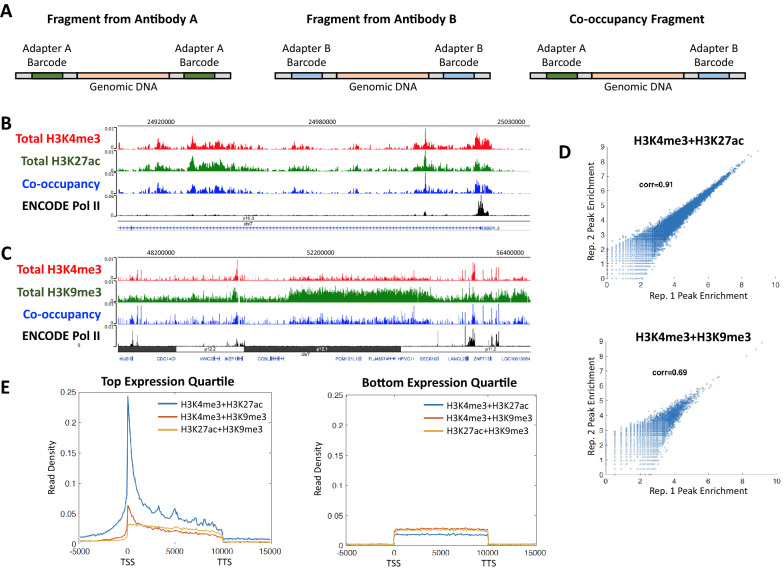
ACT2-seq directly profiles co-occupancy between pairs of epigenetic factors. **A** Illustration of the types of DNA fragments generated by the ACT2 method. Each fragment type provides enrichment information for either antibody A, antibody B, or both (co-occupancy). **B** Genome browser snapshot of a representative genomic region enriched for “active” chromatin marks. The top three tracks were obtained from a single sample in which H3K4me3 and H3K27ac were mapped concurrently. Four data tracks are provided: one for total H3K4me3 signal, one for total H3K27ac signal, one for only co-occupancy reads, and one for ENCODE ChIP-Seq of RNA Pol II for comparison. **C** Genome browser snapshot of a representative genomic region enriched for both H3K9me3 and H3K4me3. The top three tracks were obtained from a single sample in which H3K4me3 and H3K9me3 were mapped concurrently. Four data tracks are provided: one for total H3K4me3 signal, one for total H3K9me3 signal, one for only co-occupancy reads, and one for ENCODE ChIP-seq of RNA Pol II for comparison. Note the region of high H3K9me3 enrichment that roughly correlates to chromosome band p12.1 in black. **D** Scatter plots examining peak enrichment correlation between two biological replicates of co-enrichment data. Each data point represents the read density of a single enriched region. **E** Metagene profiles of average read density within annotated genes for co-enrichment of the indicated epigenetic factors. All three co-enrichment data sets were obtained from a single concurrent mapping of H3K4me3, H3K27ac, and H3K9me3. TSS: transcription start site. TTS: transcription termination site. Gene lengths have been scaled to 10 kb to facilitate alignment. Plots are provided for gene sets representing the top and bottom expression quartiles

In addition to concurrently mapping multiple epigenetic factors, ACT2 simultaneously probes co-occupancy of the factors across the genome. This capability arises from the use of oligonucleotide adapters with distinct barcodes for each enzyme-antibody complex. For example, when using two antibodies “A” and “B”, three distinct configurations of barcodes could be present at either end of the sequencing reads when the two factors are in close spatial proximity on chromatin (Fig. 3A). A DNA fragment with both barcodes matching the enzyme-antibody complex associated with either antibody A or B indicates a signal arising from that antibody alone. However, fragments bearing an antibody. A barcode at one end and an antibody B barcode at the other end represent a signal arising from both antibodies simultaneously. Importantly, these barcode-mismatched fragments can only arise when both epigenetic factors are present together in close proximity and thus directly indicate co-occupancy of the two factors on the same chromatin strand. Thus, ACT2 data files can be partitioned into sub-samples representing various types of enrichment or co-enrichment signals based on the barcodes of the sequence reads (see Table 1 in Methods).

**Table 1:** Data sets partitioned from an example ACT-seq sample using two antibodies

Name	Barcode 1	Barcode 2	Data type
A-only	Antibody A	Antibody A	Signal arising from antibody A and no others
A-total	Antibody A	Any	Total signal from all antibody A fragments
B-only	Antibody B	Antibody B	Signal arising from antibody B and no others
B-total	Antibody B	Any	Total signal from all antibody B fragments
Co-oc.^a^	Antibody A	Antibody B	Signal for co-occupancy of the two factors

^a^Note that samples in which more than two factors are mapped concurrently will also generate additional co-enrichment data sets for each distinct combination

To investigate the ability of ACT2 to probe co-occupancy of epigenetic factors, we used the barcode strategy discussed above to partition samples in which H3K4me3 was concurrently profiled alongside H3K27ac, H3K9me3, or both. Consistent with our previous analyses (Fig. 2), we found that co-occupancy of the active histone modifications H3K4me3 and H3K27ac resembled the shared peak patterns of both marks (Fig. 3B). In contrast, we observed many broad regions of H3K9me3 enrichment that exhibited relatively lower levels of H3K4me3. These likely represent transcriptionally repressed and/or heterochromatic chromosome domains as suggested by the overlap between the broad region of H3K9me3 enrichment and the chromosome band p12.1 found in the central region of Fig. 3C. Visual inspection of the data revealed that such H3K9me3-rich domains also exhibited relatively lower levels of H3K4me3 + H3K9me3 co-occupancy compared to most isolated peaks (Fig. 3C). To determine whether co-occupancy signals obtained using ACT2 were reproducible, we compared peak enrichment levels between biological replicates as was done in Fig. 1D. We observed very high reproducibility in peak enrichment for replicates of H3K4me3 + H3K27ac co-occupancy (Fig. 3D, top). The reproducibility of H3K4me3 + H3K9me3 co-occupancy was lower (Fig. 3D, bottom), likely due in part to the dissimilar enrichment profiles of these marks (Fig. 2A–C).

It should be noted that profiling more than two epigenetic factors in a single ACT2 sample will produce a separate co-occupancy data set for each pairwise combination of factors. For example, the sample in which H3K4me3, H3K27ac, and H3K9me3 were all probed concurrently (Fig. 2A, right) provided three co-occupancy data sets: H3K4me3 + H3K27ac, H3K4me3 + H3K9me3, and H3K27ac + H3K9me3. We constructed metagene profiles for these co-occupancy data sets at highly expressed and at lowly expressed genes (Fig. 3E). At highly expressed genes, we observed that H3K4me3 and H3K27ac exhibited elevated average levels of co-occupancy at TSSs, whereas co-occupancy of either of these marks with H3K9me3 was present at much lower levels (Fig. 3E, left). Interestingly, H3K27ac + H3K9me3 co-occupancy exhibited a distinguishable pattern from H3K4me3 + H3K9me3, suggesting the presence of different mechanisms of interaction between these pairs of epigenetic marks. In contrast, lowly expressed genes did not exhibit peaks of H3K4me3 + H3K27ac co-occupancy (Fig. 3E, right), consistent with our previous observation of expression-level-dependent distributions of these marks (Fig. 2B).

**Fig. 4:**
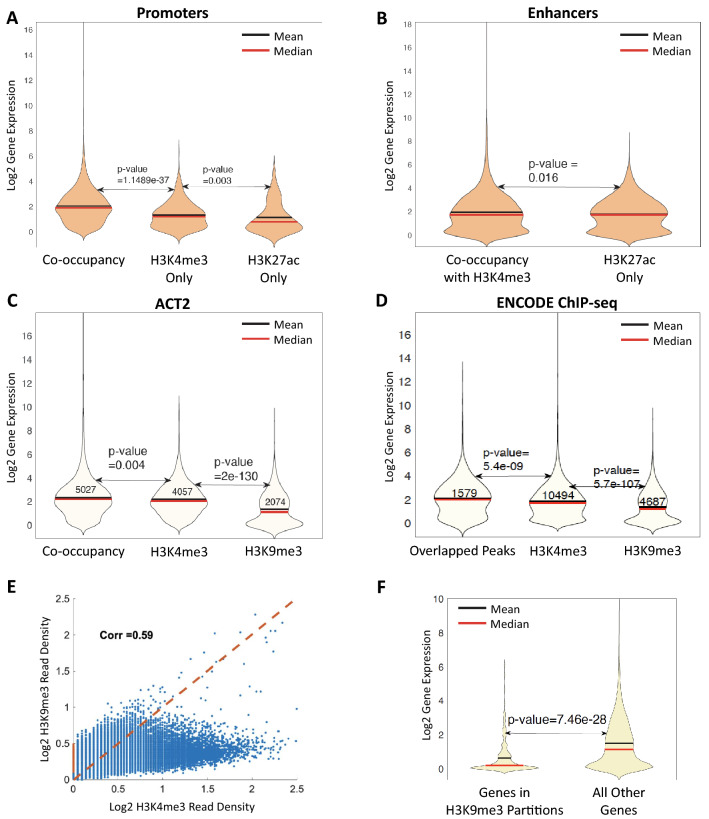
Co-enriched genomic elements identified using ACT2 exhibit distinct gene expression profiles. **A**–**D** Violin plots depicting expression profiles of gene sets in the indicated categories. *p-*values were obtained using Wilcoxon rank sum tests. **A** Violin plot depicting the expression profiles of genes that exhibited (co-)enrichment of H3K4me3 and/or H3K27ac at the gene promoter. Gene expression was calculated as reads per kilobase mapped (RPKM). Using a significance threshold of α < 0.005, the null hypothesis of no difference in the gene expression profile of co-occupied promoters was rejected. **B** Violin plot as in panel A but using enrichment status at gene enhancers. Enhancers were defined as regions enriched for H3K27ac, and thus the H3K4me3-alone category was not included. Gene expression values were matched to enhancers based on proximity. Using a significance threshold of α < 0.005, the null hypothesis of no difference in the gene expression profile of co-occupied enhancers was not rejected. **C** Violin plot depicting the expression profiles of genes that exhibited (co-)enrichment of H3K4me3 and/or H3K9me3 at the gene promoter. Gene expression was calculated as reads per kilobase mapped (RPKM). Using a significance threshold of α < 0.005, the null hypothesis of no difference in the gene expression profile of co-occupied promoters was rejected. **D** Violin plot as in panel C but using ENCODE ChIP-seq data. Since co-occupancy cannot be examined in these data, the presence of overlapping H3K4me3 and H3K9me3 peaks within the promoter regions was used as a proxy. Using a significance threshold of α < 0.005, the null hypothesis of no difference in the gene expression profile of promoters containing overlapping expression peaks was rejected. **E** Scatter plot examining the correlation between H3K9me3 and H3K4me3 read densities across the genome. Each data point represents a genomic fragment of five kilobases in length. Fragments exhibiting only H3K9me3 enrichment with no detectable H3K4me3 enrichment are depicted in red on the *y*-axis. **F** Violin plot depicting the expression profiles of genes that exhibited enrichment of H3K9me3 but not H3K4me3 at the transcription start site (TSS) vs. all other genes. Gene expression was calculated as reads per kilobase mapped (RPKM). Using a significance threshold of α < 0.005, the null hypothesis of no difference in the gene expression profile of these genes vs. all other genes was rejected

The observed co-occupancy profiles among H3K27ac, H3K4me3, and H3K9me3 at high vs. low expression genes (Fig. 3E) prompted us to examine if the presence of co-occupancy among these factors at promoter or enhancer elements was predictive of gene expression levels. Genes were separated into categories based on whether the promoter was enriched for H3K4me3 alone, H3K27ac alone, or co-occupied by both histone marks. We then compared the distributions of expression for genes in each category. We observed that co-occupancy of H3K4me3 and H3K27ac at gene promoters was correlated with a statistically significant elevation in gene expression compared to promoters enriched for either mark alone (Fig. 4A). However, the expression profile for promoters enriched for H3K4me3 alone was not significantly different from those enriched for H3K27ac alone, indicating that the expression effects arising from the presence of either mark individually are not broadly distinguishable. We performed a similar analysis of gene-proximal enhancer regions. Proximal enhancers were identified as regions enriched for H3K27ac using a distance cutoff of five kilobases from the nearest gene. Proximal enhancers were sorted into two categories based on whether they were enriched for H3K27ac alone or co-occupied with H3K27ac and H3K4me3. We did not observe a significant difference in gene expression for co-occupied proximal enhancers compared to enhancers enriched for H3K27ac alone (Fig. 4B). Together, these data indicate that co-occupancy of H3K4me3 and H3K27ac is correlated with higher gene expression levels if present at promoters, but do not broadly affect gene expression if present at proximal enhancer elements.

Interestingly, we identified a distinct class of genes that exhibited co-occupancy of H3K4me3 and H3K9me3 at the promoter (Fig. 4C). This is notable due to the broader differences in the genomic distributions of these marks (Fig. 2A–C). While this class of co-occupied promoters is correlated with marginally elevated gene expression, the effect is very small compared to co-occupancy of H3K4me3 + H3K27ac (Fig. 4C vs. A). Precedent for this effect can be found in ENCODE ChIP-seq data for these marks. While it is not possible to examine co-occupancy using ChIP-seq, we identified gene promoters containing overlapping H3K4me3 and H3K9me3 enrichment peaks as a proxy. Such dual-enriched promoters exhibited significantly elevated expression levels compared to promoters enriched for either mark alone (Fig. 4D). Together, these data support the presence of a separate class of gene promoters exhibiting H3K4me3 and H3K9me3 co-enrichment that are associated with a marginally elevated gene expression distribution. Importantly, promoters enriched for H3K9me3 alone were associated with sharply reduced expression levels in both the ACT2 and ChIP-seq data sets (Fig. 4C–D), consistent with the characterized role of this mark in transcriptional repression. Notably, direct probing of co-occupancy revealed a higher number of co-enriched genes (Fig. 4C) compared to simple overlapping of ChIP-seq peaks (Fig. 4D), suggesting that the ACT2 approach is more sensitive.

The unexpected presence of promoter co-occupancy of H3K4me3 and H3K9me3, which are normally associated with active and repressed transcriptional states, respectively, prompted us to examine genome-wide correlations between these marks in a gene- and peak-agnostic manner. We computationally sectioned the genome into five-kilobase segments and examined the read densities of H3K4me3 and H3K9me3 within each segment (Fig. 4E). In contrast to the H3K4me3 levels that have a large dynamic range (Fig. 4E, increasing *x*-axis values), the H3K9me3 level have a relatively narrow dynamic range. We examined the expression of genes associated with relatively high levels of H3K9me3 but with no or very low levels of H3K4me3 signals (Fig. 4E, highlighted in red (comprising 4,370 genome segments and 1,852 genes). We found that expression of the genes in these segments were sharply reduced relative to the larger gene population (Fig. 4F), again consistent with the repressive nature of H3K9me3 and indicating that our genome partitioning is accurately capturing the roles of these histone marks. Importantly, a small fraction of genomic segments exhibited co-linear read densities for both H3K4me3 and H3K9me3 (Fig. 4E, upper-right quadrant). In other words, a subcategory of genomic segments eluded the standard trend and exhibited elevated enrichment of both H3K4me3 and H3K9me3. Combined with the unexpected presence of H3K4me3 + H3K9me3 co-occupied gene promoters revealed using ACT2, these data suggest the existence of novel mechanisms of genomic interplay between these seemingly disparate histone modifications.

## Discussion

ACT2-seq relies on tagmentation by the Tn5 transposase subunit of the PA-Tn5 enzyme to generate DNA fragments for sequencing. This technology greatly facilitates the speed and simplicity of the ACT2 protocol but presents some technical considerations for the user. For example, generation of sequence-able genomic fragments by tagmentation requires two nearby adapter insertion events by the transposase (one for each adapter-tagged end of the fragment). If probing an epigenetic mark that is exceedingly sparsely distributed, it could prove difficult to accumulate the insertion events required to generate a substantial number of tagged fragments for sequencing. However, ACT2 may provide an advantage in this area compared to other targeted DNA-fragmentation methods. The ability to co-profile multiple epigenetic marks in the same sample presents the opportunity to introduce a larger number of tag-insertion events compared to probing a single sparsely distributed epitope alone. This could greatly increase the likelihood of generating sequence-able fragments for such epitopes.

Co-enrichment of epigenetic factors can also be investigated using single-cell methods. However, single-cell methods are much more technically complex and require methods such as drop fluidics or split-pool barcoding. In addition, even the latest single-cell protocols suffer from sparse or missing data issues that can confound co-occupancy studies or limit their use to a small number of target sites. Even under ideal circumstances, single cells generally possess more than one copy of most genes, which makes it difficult to conclusively identify co-occupancy of epigenetic factors. A primary advantage of ACT2 is the ability to directly profile co-occupancy of factors on the same chromosome strands without the need to obtain single-cell data. In addition, the simplicity of the ACT2 protocol facilitates rapid concurrent mapping of epigenomic marks and avoids the need for expensive reagents such as library preparation kits or magnetic beads.

## Conclusions

ACT2-seq was demonstrated to be capable of concurrent mapping of multiple epigenetic marks in a single sample. Further, ACT2-seq simultaneously provides co-occupancy profiles between pairs of probed marks without the need for additional experimental steps. These advantages make ACT2 an appealing alternative to currently available methods for epigenomic profiling that require such laborious procedures and/or expensive reagents. Using ACT2, we identified a strong correlation between H3K4me3 + H3K27ac co-occupancy at transcription start sites and gene expression levels. Further, we identified genomic co-occupancy of H3K4me3 and H3K9me3, two epigenetic marks that otherwise exhibit disparate patterns of enrichment. Based on its simplicity, technical advantages, and ability to profile co-occupancy of epigenetic factors, we conclude that ACT2-seq presents an appealing alternative to currently available methods for epigenomic profiling.

## Materials and methods

### Detailed protocols

Detailed bullet-point versions of the ACT2-seq and ACT-seq protocols are available at The Protocol Exchange website (https://protocolexchange.researchsquare.com/).

### Reagents

- MinElute PCR Purification Kit (Qiagen catalog # 28004).
  MinElute Gel Extraction Kit (Qiagen catalog # 28604).
  Phusion 2X HF Master Mix (New England Biolabs catalog # M0531S).
  H3K4me3 antibody (Millipore Sigma catalog # 07-473).
  H3K27ac antibody (Abcam catalog # ab4729).
  RNA Pol II antibody (Millipore Sigma catalog # 17-620).
  Rabbit IgG isotype control (Abcam catalog # ab37415).

### Public data sets

The following publicly available data sets were used to validate the ACT2 results: ENCFF375WTP (H3K4me3), GSM3965505 (H3K27ac), ENCFF782FRS (H3K9me3), ENCFF865BUP (Pol II), and GSM4156601 (RNA-seq).

### Design and preparation of the PA-Tn5 complex adapters

As in ACT-seq, the oligonucleotide adapters that are bound by the PA-Tn5 enzyme are partially double stranded after being annealed to the pMENTS (“mosaic-end, non-transfer strand”) oligonucleotide. In ACT2, however, each adapter bears a distinct barcode sequence to enable the signals from each antibody to be distinguishable in the resulting sequence data. The general sequences are provided in the following table, given in 5′ to 3′ orientation:

**Table:** 

Oligo Name	Sequence
pMENTS	5′Phos-CTGTCTCTTATACACATCT
Complex Adapter A	CCTACACGACGCTCTTCCGATCTNNNNNNNNAGATGTGTATAAGAGACAG
Complex Adapter B	TTCAGACGTGTGCTCTTCCGATCTNNNNNNNNAGATGTGTATAAGAGACAG

where the poly-N region represents the variable barcode sequences of 7 or 8 nucleotides in length. The sequences upstream (left, 5′) of the barcodes are designed to anneal to either the i5 or i7 PCR primers that are used later in the protocol during library preparation. The sequence downstream (right, 3′) of the barcodes is the mosaic end (ME) sequence that is bound by the Tn5 enzyme. We recommend varying the lengths of the barcodes between 7 and 8 bp to help promote sequence complexity in the resulting libraries. For a full list of all adapters used in this study, see Additional file 1.

Prior to use, each complex adapter was annealed to pMENTS using the following procedure: 25 μl of 100 μM complex adapter was mixed with 25 μl of 100 μM pMENTS. The mixture was heated to 99 °C for 5 min, and the heat source was then turned off and the solutions were allowed to cool to room temperature over the course of two hours or overnight. The resulting 50 μM annealed adapters were then stored at 4 °C or −20 °C until use.

### Design of the PCR library adapters

The adapters used in the library preparation steps are distinct from the complex adapters detailed above. The library adapters are based on Illumina NextEra designs and are barcoded to enable different samples to be distinguished from one another after multiplexing. The general sequences are provided in the following table, given in 5′ to 3′ orientation:

**Table:** 

Oligo name	Sequence
Library Adapter A	AATGATACGGCGACCACCGAGATCT- ACACTCTTTCCCTACACGACGCTCTTCCGATCT
Library Adapter B	CAAGCAGAAGACGGCATACGAGAT- NNNNNNNNGTGACTGGAGTTCAGACGTGTGCTCTTCCGATCT

For this study, a single universal Library Adapter A sequence was used in all samples, whereas each sample received a variant of Library Adapter B containing a unique i7 index sequence (represented by the 8 bp poly-N region in the table). This enables the samples to be distinguished from one another after multiplexing. For a full list of all adapters used in this study, see Additional file 1.

### Expression of recombinant PA-Tn5

The expression vector containing the PA-Tn5 construct is available from Addgene under accession number 121137 (http://www.addgene.org/121137/). Procedures for expression and purification of recombinant PA-Tn5 are provided in the original ACT-seq manuscript and in more detail in the bullet-point ACT2 protocol available on The Protocol Exchange.

### Preparation of the antibody-PA-Tn5 complex

Separate PA-Tn5 complexes with unique adapters were prepared for each antibody. Separate 1.5 mL tubes were labeled for each desired PA-Tn5 complex and the following was added: 4.5 μl of 2X Complex Buffer (0.1 M Tris pH 8.0, 0.3 M NaCl, 0.1% Triton X-100, 25% glycerol) supplemented with an EDTA-free protease inhibitor cocktail to 2X concentration, 2 μl of an annealed 50 μM Complex Adapter A, 2 μl of an annealed 50 μM Complex Adapter B, and 2.5 μl of ~ 1 μg/μl purified recombinant PA-Tn5 enzyme. The solutions were mixed by pipetting and incubated at room temperature for 10 min.

A fresh set of 1.5 mL tubes was labeled for each biological sample. Each tube received 1.5 μl of the appropriate complex prepared above, 1.5 μl of 2X Complex Buffer, and 1.5 μl of the matching antibody. The samples were mixed by pipetting and incubated at room temperature for 30 min. The prepared antibody-PA-Tn5 complexes can be left at room temperature prior to use for several hours if necessary. Note that we typically add the antibodies volumetrically due to the difficulty in obtaining accurate concentration measurements from many antibody manufacturers.

For probing multiple epigenetic marks in the same samples, separate aliquots of antibody-PA-Tn5 complexes should be prepared for the subsequent antibodies as well.

### Cell crosslinking and permeabilization

Cells were crosslinked by incubating for 10 min in 10 ml of culture medium supplemented with 0.25% formaldehyde. The cells were pelleted and washed twice in 10 ml of PBS. 1 million cells were transferred to a 1.5 ml tube and suspended in 1 ml of freshly prepared RIPA Buffer (150 mM NaCl, 0.2% SDS, 0.1% sodium deoxycholate, and 1% Triton X-100 in TE buffer pH 7.5) for 10 min. The permeabilized cells were pelleted at 850*g for 2 min and the supernatant was carefully removed to ~ 50 μl. The cells were suspended in 1 ml of Wash Buffer (50 mM Tris pH 8.0, 150 mM NaCl, 0.05% Triton X-100, 2 mM EDTA). The cells were pelleted as above, and the supernatant was removed to ~ 50 μl. The cell suspension was diluted to 1 ml using Wash Buffer to generate an approximate concentration of one thousand cells per microliter.

### Complex binding, washing, and tagmentation

The cell suspension was gently mixed, and 50 μl of this suspension was added to each of the sample tubes containing the first set of antibody-PA-Tn5 complexes. The samples were mixed gently and incubated for 30 min at room temperature to allow the complexes to bind to chromatin. After incubation, 1 ml of Wash Buffer was added to each sample, and the tubes were sealed and mechanically rotated for 5 min at room temperature. The cells were pelleted at 850*g, and the supernatant was removed to ~ 50 μl. At this point, 3 μl of an annealed 50 μM “blocking adapter” were added to each sample. The blocking adapter is any annealed Tn5 complex adapter that features a barcode sequence that is not used in any of the samples. The samples were gently mixed and incubated at room temperature for 10 min to allow the blocking adapter to bind to any residual PA-Tn5 complexes that did not take up their original adapters.

After incubation with the blocking adapter, the second set of prepared antibody-PA-Tn5 complex solutions were added to the matching samples. The samples were mixed gently and incubated at room temperature for 30 min. 500 ul of Wash Buffer was added to each sample, and the tubes were sealed and mechanically rotated for 5 min at room temperature. The cells were pelleted at 850*g, and the supernatant was removed to ~ 50 μl. The 500 μl wash and pelleting steps were repeated once. Each sample was diluted to ~ 100 ul using Wash Buffer and received 1.5 μl of 1 M MgCl_2_. The samples were mixed by pipetting and incubated at 37 °C for one hour to allow targeted tagmentation to occur.

### Purification of DNA fragments

The tagmentation reactions were stopped by adding 8 μl of 0.5 M EDTA to each sample. The samples were mixed by vortexing and incubated at 80 °C for 5 min to deactivate the transposase. Each sample received 2 μl of 10% SDS and 1 μl of 20 mg/ml proteinase K enzyme. The samples were incubated at 55 °C for 60 min. After the incubation, the samples were purified using a MinElute PCR Purification Kit and an elution volume of 20 μl. After purification, samples may be stored at −20 °C until library preparation is performed.

### Library preparation

The purified ~ 20 μl samples were transferred to PCR tubes for library preparation. Each tube received 0.5 μl of 50 μM universal Library Adapter A and 0.5 μl of a 50 μM Library Adapter B with a unique barcode. 20 μl of 2X Phusion HF Master Mix was added to each sample and mixed. PCR was performed to amplify libraries using the following program: 
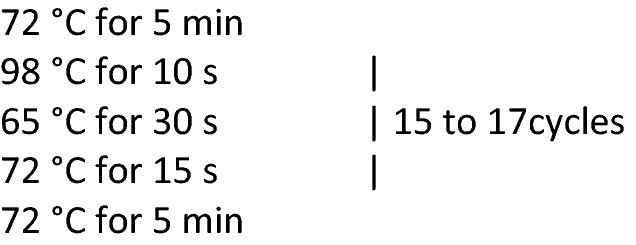


The resulting PCR products were visualized using gel electrophoresis on 2% agarose E-gels. Gel slices were excised corresponding to DNA fragment sizes between ~ 250 and 850 bp. Gel purification was performed using a MinElute Gel Purification Kit. The purified DNA libraries were quantified using a Qubit fluorometer. Libraries were sequenced on Illumina’s NovaSeq platform using PE150 format.

### Assembly of data sets from ACT2-seq libraries

Each ACT2 sample comprised data from two or three concurrently profiled epigenetic marks along with co-occupancy data. Thus, the files required partitioning prior to analysis. For an example sample in which two factors were mapped using antibodies “A” and “B”, five partitioned. fastq file pairs were generated for examination and analysis (Table 1, Additional file 3). These files were populated based on the combination of adapter barcode sequences at either end of the DNA fragments.

Reads from all sub-libraries were mapped separately to the human genome (hg19) using Bowtie2 [21]. Reads with a MAPQ quality score less than 10 and duplicate reads were removed. The specific sub-libraries used to generate each figure are noted in Additional file 2.

### Enriched regions and their annotations

Enriched regions were identified using SICER [22] with the following parameters: window size of 200 bp, gap size of 200 bp, and an E-value of 100. Enriched regions were aligned to annotated genes using the intersect function from BEDTools [23]. Enhancer elements were defined as regions of H3K27ac enrichment that were located at least five kilobases distant from an annotated TSS. The annotation of enriched genomic elements was performed using the annotatepeaks.pl utility of HOMER (http://homer.ucsd.edu/homer/).

## Supplementary Information

**Additional file 1:** Oligonucleotide adapters used in this study.

**Additional file 2:** Mapping statistics and sub-libraries used in each figure.

**Additional file 3:** Scripts used in data analysis and partitioning.

## Data Availability

All genome-wide data sets generated for this study are available at the Gene Expression Omnibus under Accession Number GSE179047.
